# Measuring the Lifespace of People With Parkinson’s Disease Using Smartphones: Proof of Principle

**DOI:** 10.2196/mhealth.2799

**Published:** 2014-03-12

**Authors:** Jacki Liddle, David Ireland, Simon J McBride, Sandra G Brauer, Leanne M Hall, Hang Ding, Mohan Karunanithi, Paul W Hodges, Deborah Theodoros, Peter A Silburn, Helen J Chenery

**Affiliations:** ^1^UQ Centre for Clinical ResearchAsia-Pacific Centre for NeuromodulationThe University of QueenslandHerston, QLDAustralia; ^2^The Australian E-Health Research CentreCSIRO Computational InformaticsBrisbane, HerstonAustralia; ^3^School of Health and Rehabilitation SciencesThe University of QueenslandBrisbaneAustralia

**Keywords:** Parkinson's disease, community, telemedicine, mHealth

## Abstract

**Background:**

Lifespace is a multidimensional construct that describes the geographic area in which a person lives and conducts their activities, and reflects mobility, health, and well-being. Traditionally, it has been measured by asking older people to self-report the length and frequency of trips taken and assistance required. Global Positioning System (GPS) sensors on smartphones have been used to measure Lifespace of older people, but not with people with Parkinson’s disease (PD).

**Objective:**

The objective of this study was to investigate whether GPS data collected via smartphones could be used to indicate the Lifespace of people with PD.

**Methods:**

The dataset was supplied via the Michael J Fox Foundation Data Challenge and included 9 people with PD and 7 approximately matched controls. Participants carried smartphones with GPS sensors over two months. Data analysis compared the PD group and the control group. The impact of symptom severity on Lifespace was also investigated.

**Results:**

Visualization methods for comparing Lifespace were developed including scatterplots and heatmaps. Lifespace metrics for comparison included average daily distance, percentage of time spent at home, and number of trips into the community. There were no significant differences between the PD and the control groups on Lifespace metrics. Visual representations of Lifespace were organized based on the self-reported severity of symptoms, suggesting a trend of decreasing Lifespace with increasing PD symptoms.

**Conclusions:**

Lifespace measured by GPS-enabled smartphones may be a useful concept to measure the progression of PD and the impact of various therapies and rehabilitation programs. Directions for future use of GPS-based Lifespace are provided.

## Introduction

### The Lifespace Construct

**Figure 1:**
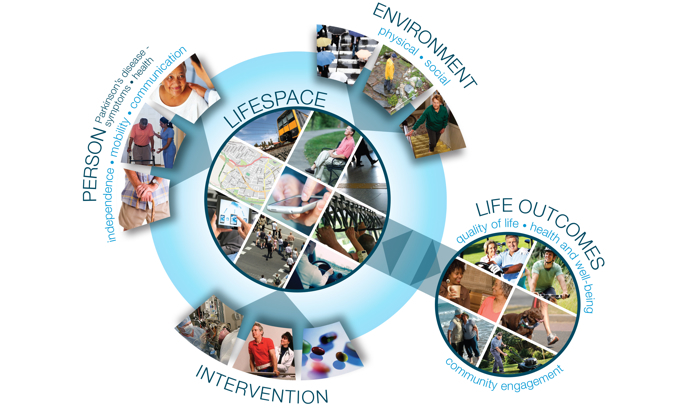
Lifespace as it relates to a person, environment, intervention, and life outcomes.

Lifespace is a measure of the geographic space in which a person lives and conducts their roles and activities [1,2], and captures the extent to which they travel and their patterns of movement within the community (Figure 1 illustrates the construct). As a construct, it arose from gerontological research, focusing attention on the relationship between the person and their environment [3,4]. It was originally conceptualized as concentric circles around the person representing expansion in the areas in which a person lived and interacted from their bedroom, to the house, and extending to the world outside the local neighborhood [1,4,5]. The research into Lifespace suggests that it is interconnected with a person’s health and functional status [6-8], their environment, including available resources [9,10] and interventions focusing on the person, their health, and/or their environment [7,8,11]. Lifespace is an indication of the broader participation and quality of life outcomes [2,12]. It is thought to represent opportunity for community participation and social interaction [2,13] and also represents actual lived function and community access over a period of time [1,2,8].

### Lifespace Individually Defined

There is no optimal amount of Lifespace. Like many aspects of quality of life and participation, it is individually defined. Individuals are likely to have different desired patterns and locations of community engagement [8,12]. The research into frail, community-dwelling older people in Japan has suggested that it is desirable that people leave their homes and engage in their neighborhood at least once a week to maintain function and health [14]. Xue et al [15] suggested that leaving the home less than four times a week was a risk factor or marker for future frailty, regardless of the way in which community mobility occurred or if assistance was required.

As Lifespace is a construct that fits within the person-environment and World Health Organization’s International Classification of Functioning models, it is able to indicate the broad, enacted impact of health and environment on participation [16]. The research has shown that Lifespace can demonstrate the frequency and distance of independent community mobility or supported travel involving equipment or assistance from others, and therefore could be used to evaluate the impact of physical, cognitive, psychiatric, and sensory symptoms on life participation [1,5]. In addition, Lifespace is a construct that can capture how the design and resources available to an individual could enable high levels of participation and large geographic areas of engagement, even in the face of substantial symptomatology or disability. Indeed, optimal design of communities and resource allocation enable maintained Lifespace [16,17].

Reflecting its development in gerontology, Lifespace has been most widely applied in studies of the impact of ageing for people living in the community, for example, reference [15]. The longitudinal studies have indicated that even subtle restrictions in Lifespace can indicate prodromal changes in relation to illness and ageing, including future onset of functional deficits, for example, reference [14], cognitive impairment [18], and mortality [15]. Some studies on Lifespace have also described the differential adjustment to community life after hospitalization [7], the impact of falls [17], and the impact of roles including caregiving, for example, reference [19]. A number of the studies involving populations of older people have identified that illnesses or disorders affecting mobility have a direct impact on Lifespace, and have specifically noted Parkinson’s disease (PD) as affecting Lifespace, for example, references [9,14].

### Lifespace and People With Parkinson’s Disease

Lifespace has not been extensively studied for people with PD, although outdoor walking and community mobility have been researched, for example, reference [13]. It has clear relevance as the motor (difficulties with gait, tremor, and rigidity) and nonmotor symptoms (apathy, depression, sleep disturbance), as well as the most common reasons for hospitalization, (falls and psychiatric symptoms), could impact on a person with PD’s desire and ability to regularly access the community [20]. Some studies into community walking for people with PD have highlighted the complex challenges associated with outdoor mobility. The impact of symptoms, confidence, and personal strategies, as well as environmental barriers, combine to impact on the community participation of people with PD [13,21]. The symptoms of PD have been shown to have variability over the short term within the overall trend of slow progression of the disease, rapid and unpredictable changes to symptoms may be seen. This has led to increased interest in monitoring the ongoing symptoms and outcomes for people with PD in their home environments [22].

Interventions for people with PD can target safe outdoor mobility, confidence with mobility, and ease with leaving the home [23,24]. Lifespace therefore gives an opportunity to monitor the implications of the combined impact of the symptoms of PD, the available supports, and the impact on daily life. Caregiving for someone with PD has been identified in a qualitative study as restricting Lifespace for the carer [19], indicating the broad impact of the illness and the potential utility of the approach for monitoring the quality of life and community participation for both people with PD, and their families.

As a global measure of community participation, Lifespace also has the potential to indicate the overall impact of treatments. As well as monitoring the impact of changes to symptoms on community participation, it can also capture the broader impact of the treatment itself. Qualitative investigation of the impact of PD and its treatments has indicated, for example, that medications requiring a strictly timed regime can result in surrendering of valued activities and roles because leaving the house needs to be arranged around medication requirements [25]. Also, intensive rehabilitation programs requiring frequent hospital-based attendance may affect patterns of Lifespace. Monitoring Lifespace may therefore serve to give an overall indication of the progression and nature of symptoms, complications, and impacts of treatment, and impact on the family and community as a whole.

Historically, Lifespace has been measured by self-report, or in an institutional setting, as reported by a staff member [1,4,5]. It has been operationalized using: (1) distances travelled, either as a direct measurement or as zones (eg, local neighborhood), (2) the frequency of travel to these destinations over the nominated period of time (eg, two weeks), and, if the focus is on independence in mobility, (3) the requirement of mobility devices or assistance from another person [1,4]. Accurately recalling and noting episodes of leaving the home over the period of a week or longer can be effortful and inaccurate, and, like all measures using self-report, can also potentially be affected by a social desirability bias [23,26]. In a study of community walking involving 50 people with PD, findings indicated that when asked to report their community mobility, 64% substantially overestimated and 10% substantially underestimated their community walking compared to accelerometer recorded walking [23]. An autonomous option for measuring community mobility is therefore warranted. The use of technology to measure Lifespace is an area of recent attention in the gerontology and health literature [2,26,27]. As global positioning system (GPS) sensors are available on most smartphones, and these are a relatively inexpensive and accepted form of technology, their potential use in monitoring Lifespace is being explored with different groups, for example, reference [2].

This study aims to examine whether GPS data collected passively on a smartphone can be used to give an indication of the Lifespace of people with PD. Using the available sample, the specific aims were: (1) to investigate whether Lifespace measured using GPS data could differentiate between people with PD and the control group, and (2) to explore whether symptom severity in PD relates to Lifespace measured by GPS.

## Methods

### Source of the Data

Data used in the preparation of this article were obtained from the Michael J Fox Foundation Data Challenge [28].

Information sheets were provided to and informed consent was obtained from all participants. Ethical approval was obtained from the Massachusetts Institute of Technology Committee on the Use of Humans as Experimental Subjects (Approval number 1105004497) and The University of Queensland Medical Research Ethics Committee (Approval number 2013001470). Participants were a convenience sample of people living in the community who had PD, or were a control participant approximately age and gender matched to the PD group. Participants were made aware of the study through Parkinson’s disease support and research related networks and snowball sampling was used. Participants needed to be able to manage the smartphone and a custom built Android application (App) in terms of charging the device and turning the App on and off. Participants with PD needed to have been diagnosed for at least one year. Participants were provided with an Android smartphone and written instructions about how to use the device, and were requested to carry it on their person for at least 4-6 hours per day (a charge cycle) over a period of at least 8 weeks. Participants were also requested to charge the device overnight, then at the beginning of the day, turn on the device and the App, and conduct their normal regular activities. When the phone battery was low on power, the phone would vibrate and the participants were asked to recharge it. They were asked not to use the device when they were asleep, bathing, or swimming. The technology used to record from the sensors employed a custom built Android App that utilized the embedded sensors on the device. Data were streamed to a Web-based server via the Internet. Basic demographic information was collected from all participants, and participants with PD also completed and returned a questionnaire containing two partial subscales of the self-report section of the Unified Parkinson’s Disease Rating Scale (UPDRS) indicating the impact of motor and nonmotor symptoms on experiences of daily living at the beginning and end of the data collection period [29]. The data were collected between December 2011 and March 2012. Higher scores on the UPDRS indicate a greater impairment/disability for the participant. Included on the UPDRS were four nonmotor items (eg, cognition and apathy) and 13 motor items (eg, freezing of gait, difficulty with swallowing). Participants noted the presence and severity of these symptoms on a five point scale, with higher scores indicating more severe symptoms. The sensors on the phone collected a range of data including audio, accelerometry, and GPS. GPS data were collected while the phone was on and GPS signal was available. Only GPS data were used in the analyses reported in this study.

### Global Positioning System Data

GPS sensor readings were available as part of the large dataset and were arranged by participant. The GPS dataset provided the longitude and latitude coordinates in one second intervals with corresponding time and date stamps. Initial analysis of this data involved plotting the individual points in the form of a two-dimensional scatter plot where the x- and y-axes were the respective latitude and longitude scales [30]. Further analysis involved creating visual representations of the Lifespace of participants over the data collection period and creating metrics of Lifespace. These metrics included the furthest distance travelled, mean daily distance, percentage of recorded time at home, and the frequency of trips to-and-from-home. The GPS data were treated as a time-series data. The distance from the home to a particular GPS (latitude, longitude) point was computed using the Haversine formula. This formula computes the distance between two points described by a latitude and longitude coordinate, time and date stamps were then used to calculate daily distances. The metrics either relied on the complete GPS dataset, or data that was segregated into days. “Home” was established mathematically as the statistical mode of recorded latitude and longitude points (ie, the longitude and latitude coordinates at which the most time was recorded). No information about the actual home address of participants was available to validate this approach. Due to limitations in the accuracy of GPS data, being at home was operationalized as being within 500 meters of the mathematically established home. To compute the frequency of trips to-and-from-home metric, a mathematical model referred to as finite-state machine (FSM) was implemented in the Python programming language. This model was defined as having two possible states: (1) subject is at home (SH), and (2) subject is not at home (SNH), transitioning between each state was determined by the corresponding GPS data. For each participant, the model stepped through each GPS coordinate, if the subject was seen to be more than 500 meters from their designated home and the current state of the FSM was set to SH, the FSM transitioned to a SNH state, and the date and time stamp of the current GPS recorded. Conversely, if the current state of the FSM is SNH, and the subject is observed to be less than 500 meters from home, the FSM transitions to a SH state, and the time and date stamp recorded. To minimize false trips that may be an artifact of the inaccurate GPS data, trips measuring less than 15 minutes were ignored.

The Lifespaces of participants with PD and control participants were compared visually and statistically. Participants with PD were then ordered according to reported symptom severity and Lifespace metrics, and visually examined. Due to the small sample size, nonparametric statistics were used. STATA software (version 12SE) was used for these statistical comparisons.

## Results

### Participant Data

Data were available from 9 participants with PD and 7 control participants. Participants lived within two states in the United States, Maine (14) and California (2). There were 12 that lived in metropolitan areas, and 4 lived in semirural regions. A summary of the basic demographic information and amount of data for each group is in Table 1. In the control group, 3 participants reported no health conditions, and 4 reported health conditions including endocrine and cardiac conditions. There were 5 of the participants with PD that reported no comorbidities, with the remaining 4 reporting between one and three comorbid conditions including cardiac, oncological, opthalmological, and renal conditions. The 9 participants with PD reported a median of 9 years since diagnosis, ranging from 2 years to 20 years. Initial scores on impact of motor symptoms ranged from 5 to 23 (median 9) out of a possible 39, and for nonmotor ranged from 0 to 6 (median 2) out of a possible 12. Scores at the end of the data collection period stayed reasonably stable, with 1 participant not completing this measure, and only 2 participants showing a change greater than one point (in both cases, a decrease in symptoms).

**Table 1:** Demographic characteristics of participants.

	n	Age years, median, (IQR^a^)	Gender, n (%) male	Days recorded median, (IQR)
PD group	9	55 (55-65)	9 (78)	40 (20-64)
Control group	7	57 (53-77)	5 (71)	22 (13-47)

^a^IQR= interquartile range

### Limitations of the Global Positioning System Data

**Figure 2:**
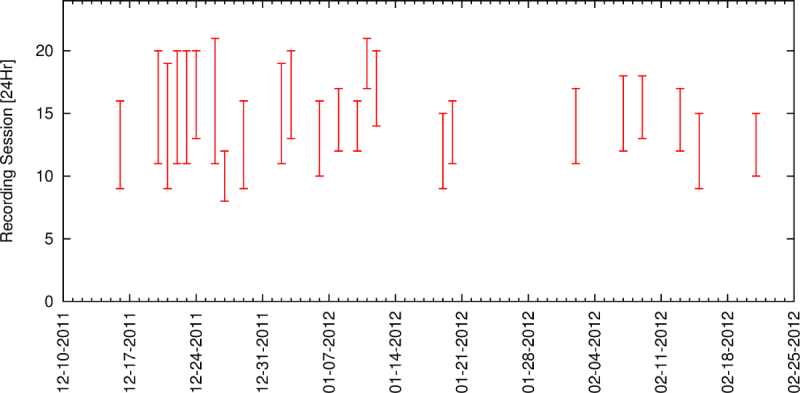
Recording periods of a participant. (Participant 3, Parkinson's disease, Aged 57).

Examination of the data revealed that the time periods represented in the dataset (days and times of day when the phone collected data) were not consistent, and also did not cover the full day (see Figure 2 shows the recording times). This should be taken into account when considering the data. Information about neighborhoods and zones, as they related to participants, was not available, and neither was the amount and type of assistance required for community trips. Information about in-home mobility was also not available due to the insufficient accuracy of the GPS sensor to give reliable results. GPS accuracy is dependent on the receiver sensitivity, the size of the antenna, the number of scattering objects between the GPS transmitter and the smartphone, and the number of visible, operating GPS satellites. Although the Android operating system is capable of recording accuracy, this was not recorded in the provided dataset. Experiments performed by the authors showed GPS error is significant when used indoors, and thus, the inferred information about in-home mobility is likely very limited.

### Lifespace Comparisons of Participants With Parkinson’s Disease and the Control Group

**Figure 3:**
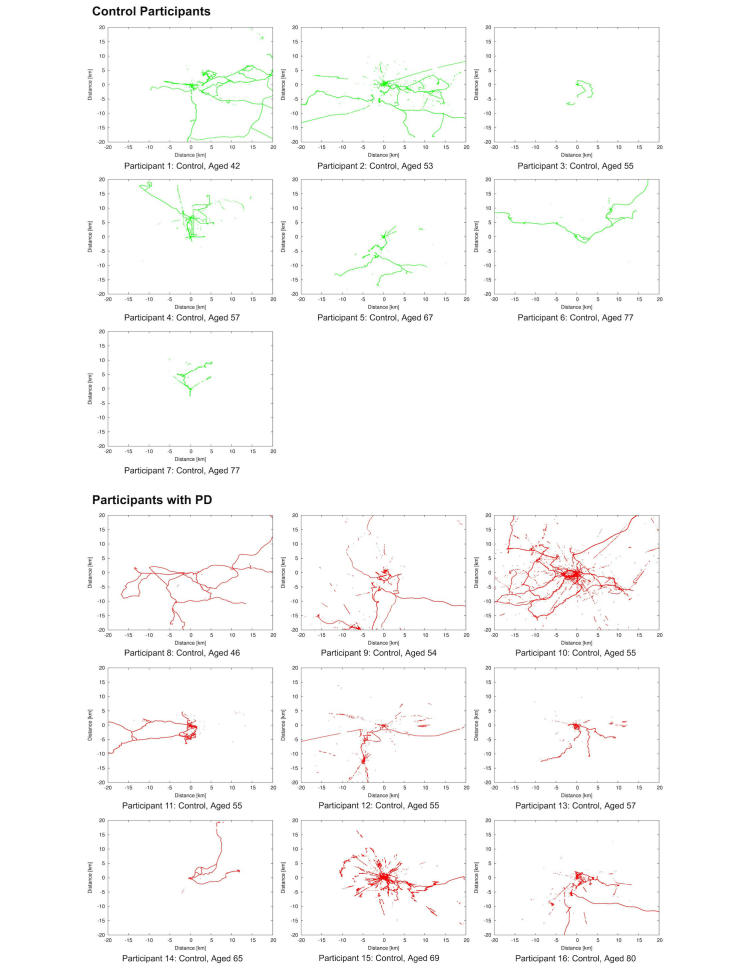
Scatterplots of Lifespace over recording period-Participants in Parkinson's disease and control groups in order of age.

**Figure 4:**
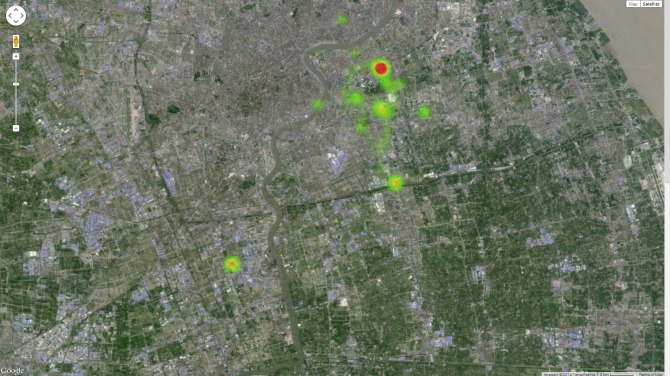
Illustrative heatmap of Global Positioning System (GPS) based Lifespace for the period of 1 week.

Figure 3 shows the visual representations of Lifespace that were created using scatterplots of the mean-subtracted latitude and longitude. To ensure the privacy of participants, scatterplots were produced using the mean-subtracted latitude and longitude coordinates rather than the raw values. The visual representations indicated that the control group was reasonably diverse in its Lifespace patterns. The Lifespace metrics were also compared between groups in Table 2. Daily Lifespace heatmaps were made for a member of each group, and the progression was visually compared (see Multimedia Appendix 1). An example heatmap, using local GPS based Lifespace data transposed into another location (in Shanghai), illustrates the nature of Lifespace heatmaps (Figure 4 shows this heatmap). Statistical testing using a Mann-Whitney *U* test indicated no significant difference between the Lifespace metrics for the control group and the PD group.

**Table 2:** Comparison of Lifespace of PD group and control group.

	Median maximum distance (km), (IQR^a^)	Median daily average distance (km), (IQR)	% of time at home, (IQR)	Median of average number of trips each week, (IQR)
PD group	67 (27-664)	10 (8-17)	56 (27-66)	6.07 (5.25-8.96)
Control group	215 (23-1056)	12.5 (7-14)	42 (26-58)	3.97 (3.50-7.89)
Statistical comparison	z=0.05 *P*=.96	z=0.21 *P*=.83	z=0.85 *P*=.40	z=1.32 *P*=.19

^a^IQR= interquartile range

### Lifespace and Severity of Parkinson’s Disease Symptoms

**Figure 5:**
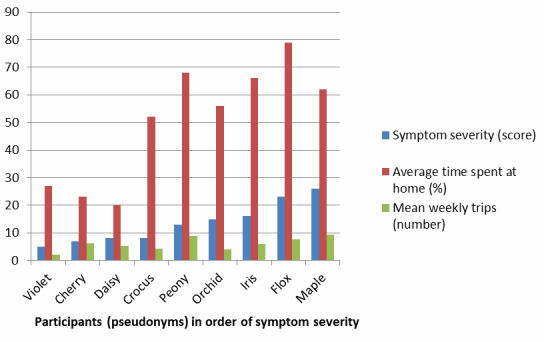
Symptom severity and Lifespace metrics for participants with PD.

To explore the relationship between the severity of PD symptoms at baseline and Lifespace, the metrics were visually compared based on symptom severity (Figure 5 shows this image), indicating a trend of decreasing Lifespace with increasing severity of reported symptoms as measured by the initial partial UPDRS score. Due to the participant numbers and distribution of scores, statistical testing was not conducted.

## Discussion

### Global Positioning System Data From Smartphones Can Indicate Lifespace

This study indicated that Lifespace might provide an objective outcome measure embedded in the person’s local context that is useful for monitoring the lived community access and participation of people with PD. This study used a released dataset to explore whether Lifespace could be meaningfully identified and analyzed from passively collected GPS data from smartphones. As a proof of principle, the findings of this study indicate that GPS data can be analyzed to give visual representations, metrics, and may relate to the self-reported severity of PD symptoms.

### Lifespace Metrics

The Lifespace metrics showed expected trends for people with more symptoms spending additional time at home and travelling shorter distances, indicating a more constricted Lifespace. This finding is also supported by other studies that investigated the Lifespace of older people, and indicated the strong impact of movement disorders such as PD on Lifespace outcomes, for example, references [9,14].

The use of passively measured Lifespace data to monitor outcomes for PD may have a number of applications. As has been done in establishing early or prodromal periods in cognitive impairment, for example, reference [15], monitoring Lifespace may help to better understand and predict the onset and course of PD. Another application may be in investigating the impact and effectiveness of interventions for PD. Medical and surgical treatments for PD have often been monitored at the symptom or symptom-related quality of life level, for example, reference [28], but they can have broader impacts on participation. Some recent published trials and reviews of rehabilitation programs for PD have suggested the need for measurement of lived outcomes in a way that is not arduous for participants who may have cognitive and physical impairments [24,31].

### No Statistical Differences in Lifespace Between Parkinson’s Disease and Control

The current study indicated that people with PD could not be statistically differentiated from approximately age and gender matched controls on the basis of Lifespace alone. This is not a surprising finding as Lifespace is not an illness or symptom specific measure and can be affected by numerous characteristics of the person, their environment, and the supports that are available to them. The control group data indicated quite varied Lifespace metrics. Other Lifespace studies have indicated the impact of ageing, for example, reference [5], illnesses, for example, reference [32], different forms of treatment [7], and life roles including caregiving, for example, reference [19] in creating a restricted Lifespace. Based on this, it seems likely that a heterogeneous group like the control group would have diverse Lifespaces. It should also be noted that the control group appeared quite different from the PD group in how much data they recorded. The PD group chose to have their phones actively recording for more days and longer periods than the control group. This possibly reflects greater interest or higher motivation on the behalf of the participants with PD, due to the focus of the study. Due to the potential bias towards active parts of the day or week, the Lifespace findings from the recorded periods cannot be directly extrapolated into the nonrecorded time periods, and as such, cannot be easily compared across groups with different amounts of data collected (such as the control group).

### Established and New Ways to Report Lifespace

This study combined established and new ways to report Lifespace. The most commonly used metric for reporting Lifespace requires calculating a score based on frequency and assistance required for movement within specified zones (eg, home, local neighborhood), for example, reference [1]. This information was not available from the shared dataset, so this score could not be approximated. Because of this, different metrics and visual methods of representing Lifespace were therefore developed. The approach for creating scatterplots of latitude and longitude coordinates used in the current study has been previously used in exploring GPS data to give insights into Lifespace, for example, reference [33]. The other metrics analyzing GPS data mathematically to determine the location of “home”, relate time spent away from this location, and trips away from this location were new developments in Lifespace research. Future research should explore the validity of this metric, comparing it to established methods of measuring time use and Lifespace.

### A Comparison of Study Findings

A comparison of the findings of the current study can be made with Lifespace and time use studies. Xue et al [15] used a categorization of Lifespace restriction over a week, severe (never leaving home), moderate (leaving home, but never leaving the neighborhood), slight (fewer than four trips away from the neighborhood), and no restriction (leaving the neighborhood more than four times). While neighborhood zones could not be established from the GPS data in the current study, it is possible that some participants were moderately restricted, some slightly restricted, and others would show no restriction. No participants recorded zero episodes of leaving home. Time use studies use self-reported time diaries to account for how time is spent over a week. A study of 195 older community-dwelling people in Australia, which was validated against the national time use statistics, found that, when measured over a week, older people spent about 85% of their time at home, and recorded eight episodes of leaving the home [32]. The findings from the current study show much higher proportions of time spent out of home and fewer trips, but this is likely to reflect the different participant groups and methodology. The current study did not record over a full day, instead, recording for a single charge cycle when participants turned on the phone. As participants chose when and how often to switch the smartphones on, this is likely to affect the comparative representativeness of the Lifespace data. It is possible that people were more likely to switch on the smartphone when they were going out, and therefore the percentage of recorded time spent at home reflects this more active portion of their life. Future studies, using a more set recording protocol, will enable this comparison to be explored more fully. Further investigation will also be needed to compare the smartphone-based method of recording Lifespace with validated approaches for measuring Lifespace and time use.

### Participants and Transferrability

The use of a shared database, which provided limited information about the process of recruitment or the demographic information about the small number of participants, means that it is difficult to establish whether the findings from this study would be transferrable to other contexts. As a proof of principle, it seemed reasonable that the participants represented the general age range and gender profile as larger studies of community-based people with PD, for example, reference [34]. They represented a range of severity of PD, and the length of disease varied between participants, indicating that this approach may be useful throughout the duration of the disease for community-dwelling people with PD.

### Using Smartphones to Measure Lifespace via Global Positioning System Data

The use of a smartphone App to passively measure GPS is a promising approach for measurement of Lifespace. Although it could be argued that dedicated GPS loggers would provide for more accurate GPS recording due to their dedicated electronics and longer battery life, the ubiquitous nature of smartphones makes this an accessible and acceptable technology to consumers. Their inclusion of other sensors within the smartphone such as accelerometer, gyroscope, and compass, that can be used in future work, makes the choice of smartphones as the data logger of choice more appealing. Local testing using the GPS logger on a typical smartphone indicated that it has negligible effect on the battery status and the operation of the phone.

### Limitations and Future Directions

**Figure 6:**
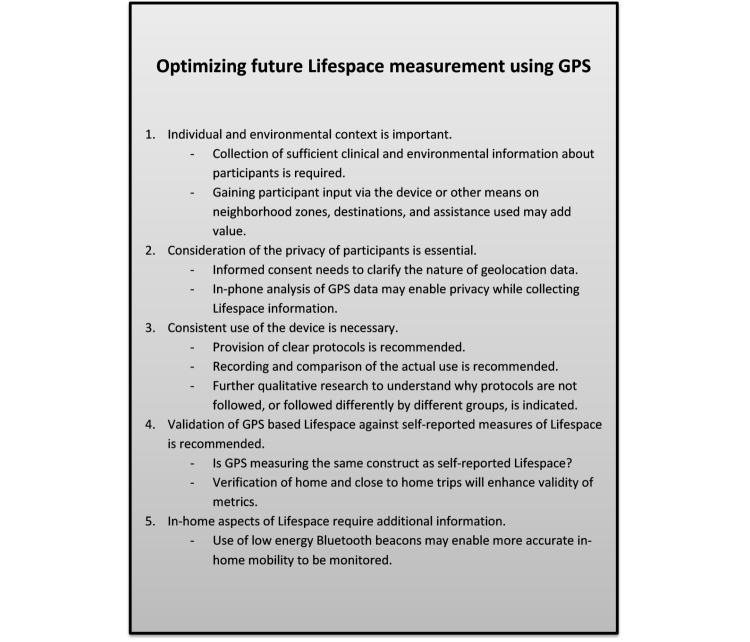
Future directions for use of GPS to measure Lifespace.

This study aimed to provide a proof of principle that GPS data collected passively via smartphones could be used to indicate the Lifespace of people with PD. It also enabled exploration of the limitations and issues encountered in measuring Lifespace via GPS in a clinical population. Some recommendations for future directions have been made (Figure 6 shows the recommendations). Despite the potential usefulness of Lifespace for monitoring the community engagement of this population, a number of limitations in the current study are discussed with particular reference as to how they might be overcome in future research. As well as using a small convenience sample that may not be representative, insufficient information was available about the objective health of or context (eg, local community resources, geography, climate) for participants, which may all be expected to influence Lifespace. The actual location of home could also not be verified. In addition, there was not a consistent protocol followed in terms of times when the smartphone collected data. This may affect the validity of the collected data in reflecting the lived experience and in enabling meaningful comparison between participants. The Lifespace metrics could not be validated against self-report measures of Lifespace due to limited information about in-home mobility, assistance required for community mobility, and reasons for and satisfaction with community travel. While the use of a partial section of the UPDRS which utilized self-report of symptoms rather than clinical evaluation reduced participant burden and study costs through not requiring clinical assessments, it is also likely to be less valid than a full clinical evaluation.

A further limitation of this approach was in the provision of GPS data in latitude and longitude coordinates. While this approach enabled accurate measuring of participants’ movements, it also revealed their locations at home and in community destinations. Care needs to be taken in collecting and sharing data of this nature or in clarifying with participants the type and detail of data collected. In the current study, the anonymity of participants was preserved in presenting the results via the calculation and reporting of mean-subtracted latitude and longitude. In recording GPS positions, having information about position accuracy is important. The accuracy of the reported latitude and longitude values can vary depending on the number of GPS satellites visible by the sensor, whether the sensor is located indoors or outdoors, ambiguities due to atmospheric changes, and interfering signals. Although the Android operating system has the capability of determining the accuracy of the GPS reading, this was not recorded and this is a limitation of the current data. To account for this, a cautious threshold of 500 meters for determining whether the person was at *home* was set. However, assuming the GPS sensor was located outdoors, it was quite likely to obtain an average accuracy of approximately 10 meters based on testing completed by the authors. Future research should record GPS accuracy to enable more valid assumptions to be made. A consequence of GPS logging is the invasion of privacy. It is expected that not all consumers or research participants would be comfortable with this. As an alternative, it is possible to log the current cellular tower to which the participant's phone is connected. While this would give coarser information on subject location and travels, it would be less intrusive and not rely on the embedded GPS sensor. The measurement of Lifespace is also complicated when participants are indoors, as the accuracy of reported latitude and longitude values degrades in this setting, as described earlier. Future work could include the use of low energy (or low power) Bluetooth beacons for more accurate indoor monitoring. We believe this will be a low cost approach and allow the smartphone to become the primary Lifespace recording device.

Future research can expand on this work through the investigation of the Lifespace of a larger group of community-dwelling people with PD and the collection of more objective baseline data about their health and local context. In addition, subjective information about their trips (eg, purpose of trip, satisfaction) could also be collected. More consistent recording of Lifespace could be enabled through the use of specific recording protocols that detail the time and duration of smartphone use. The use of smartphones could be further developed to enable better monitoring of in-home mobility and protection of the privacy of participants by allowing them to opt in and out of data collection, and converting data to a different form for analysis and sharing. The validity of the Lifespace metrics could be compared to established measures of Lifespace, time use and participation. Future research could also investigate the relationship between Lifespace and other outcomes for people with PD including presence of particular symptoms, mood, activity participation, and quality of life. The relevance of Lifespace as a measure of the effectiveness of therapeutic interventions should be further explored.

### Conclusions

This study provides a proof of principle that Lifespace is a relevant concept for monitoring the community access of people with Parkinson’s disease. Further, it can be collected passively through smartphones. While the Lifespace data collected over a period of two months did not statistically differentiate between people with PD and a control group, it did point to a relationship between the severity of reported PD symptoms and Lifespace. Measuring Lifespace using GPS-enabled smartphones may be an economical and user friendly option to measure the community access and participation of people with PD, but further research within a more robust experimental design is required.

## Multimedia Appendix 1

Video comparing daily Lifespace of a participant with PD and a control participant.

## Multimedia Appendix 2

Authors' broad video response to the Michael J Fox Foundation Data Challenge.

## Abbreviations

App: application
FSM: finite-state machine
GPS: Global positioning system
PD: Parkinson’s disease
SH: subject is at home
SNH: subject is not at home
UPDRS: Unified Parkinson’s Disease Rating Scale
